# Optimization of a rapid, sensitive, and high throughput molecular sensor to measure canola protoplast respiratory metabolism as a means of screening nanomaterial cytotoxicity

**DOI:** 10.1186/s13007-024-01289-x

**Published:** 2024-10-30

**Authors:** Zhila Osmani, Muhammad Amirul Islam, Feng Wang, Sabrina Rodrigues Meira, Marianna Kulka

**Affiliations:** 1 Faculty of Medicine, University of Alberta, Edmonton, Alberta Canada; 2 Quantum and Nanotechnologies Research Centre, National Research Council Canada, University of Alberta, 11421 Saskatchewan Drive, Edmonton, Alberta T6G 2M9 Canada

**Keywords:** Fluorescence, Absorbance, Cell viability, Protoplast, Resazurin, Canola

## Abstract

Nanomaterial-mediated plant genetic engineering holds promise for developing new crop cultivars but can be hindered by nanomaterial toxicity to protoplasts. We present a fast, high-throughput method for assessing protoplast viability using resazurin, a non-toxic dye converted to highly fluorescent resorufin during respiration. Protoplasts isolated from hypocotyl canola (*Brassica napus* L.) were evaluated at varying temperatures (4, 10, 20, 30 ˚C) and time intervals (1–24 h). Optimal conditions for detecting protoplast viability were identified as 20,000 cells incubated with 40 µM resazurin at room temperature for 3 h. The assay was applied to evaluate the cytotoxicity of silver nanospheres, silica nanospheres, cholesteryl-butyrate nanoemulsion, and lipid nanoparticles. The cholesteryl-butyrate nanoemulsion and lipid nanoparticles exhibited toxicity across all tested concentrations (5-500 ng/ml), except at 5 ng/ml. Silver nanospheres were toxic across all tested concentrations (5-500 ng/ml) and sizes (20–100 nm), except for the larger size (100 nm) at 5 ng/ml. Silica nanospheres showed no toxicity at 5 ng/ml across all tested sizes (12–230 nm). Our results highlight that nanoparticle size and concentration significantly impact protoplast toxicity. Overall, the results showed that the resazurin assay is a precise, rapid, and scalable tool for screening nanomaterial cytotoxicity, enabling more accurate evaluations before using nanomaterials in genetic engineering.

**Supplementary Information:**

The online version contains supplementary material available at 10.1186/s13007-024-01289-x.

## Introduction

Genetic engineering of plants using nanomaterials is a versatile and precise method of developing new plant cultivars for food production. One of the principal challenges of this approach is that many nanomaterials are reactive and toxic to cells at concentrations necessary for efficient transformation [1–4]. The cytotoxic effects are influenced by various factors, including nanoparticle type, size, shape, exposure duration, and the specific plant species involved [3, 4]. Additionally, nanoparticles can trigger reactive oxygen species (ROS) and cause hormone imbalances, impacting plant cell growth, germination, and overall development [5–7]. Thus, carefully considering and managing nanoparticle properties and concentrations are crucial for mitigating their toxic effects while achieving desired outcomes. Conventional methods of identifying protoplast viability include visual examination under light or fluorescent microscope, identification of genomic damage using the comet assay, or respiratory metabolism measurements using electrodes [8–10]. Although effective, these methods are technically challenging, require specialized instrumentation, and are labor intensive [11]. Traditional membrane integrity assays use staining agents such as neutral red and fluorescein to assess cell viability. In contrast, dyes such as Evans blue and trypan blue indicate dead cells by staining their contents due to membrane rupture. However, these methods can suffer from drawbacks such as background staining, limited sensitivity, and potential interference with cell function [12, 13]. The SYTOX Blue assay, which specifically stains cells with compromised membranes, effectively measures necrotic or late-apoptotic cells but may be influenced by autofluorescence from the assay components [14]. There is a need to accurately measure plant cell viability without inducing toxicity during nanomaterial-mediated transfection. Viability broadly refers to a cell’s ability to maintain metabolic activity and generate biomolecules. Few methods currently provide accurate and rapid measurement of protoplast metabolic activity in situ.

Resazurin (7-hydroxy-3 H-phenoxazin-3-one 10-oxide), is a weakly fluorescent blue dye that is reduced to highly fluorescent pink-colored resorufin (7-hydroxy-3 H-phenoxazin-3-one) in a redox reaction that involves the transfer of electrons from the reductive species to the dye molecule. This reaction is catalyzed by endogenous reductases present in cells, including mitochondrial and cytoplasmic enzymes [15]. The rate of resazurin reduction not only indicates cell viability but also reflects the overall metabolic activity of the cells. Thus, the resazurin assay effectively quantifies metabolic activity and differentiates between live and dead cells [16, 17]. This makes it a valuable tool for assessing cellular health and function. Furthermore, resazurin is water-soluble, stable in culture medium, and can penetrate cell membranes [18]. Resazurin is also non-toxic to cells, especially in low concentrations and with short exposure times [19]. The resazurin reduction assay has been employed across a diverse range of cell types and organisms, including bacteria [13], mycobacteria [20], fungi [21], suspension cells from tomato plants [22], as well as mouse and human lymphocytes [18, 23]. In plant research, this assay has been utilized for various applications, such as screening plant extracts for antibacterial activity [24], evaluating the viability of fungal spores [25], and identifying plant-derived agents against Mycobacterium tuberculosis [26]. Given its established use for assessing cytotoxicity, proliferation, and metabolic responses in human cells exposed to nanoparticles [27], we hypothesized that a similar approach could be effectively applied to plant protoplasts.

Different cell types, including plant cells from various species, have unique metabolic profiles, including variations in energy metabolism, redox status, and enzymatic activities [22]. Therefore, optimizing the resazurin reduction assay for different protoplasts is essential to ensure accurate and reliable results without compromising cellular physiology or integrity. However, there are some challenges with this approach since protoplasts are fragile and are particularly sensitive to their growth environment, especially when freshly isolated, due to their lack of a cell wall [28]. This sensitivity includes reactions to nanomaterial treatments, which can vary depending on the incubation time and concentration of nanoparticles [29]. Given the absence of a cell wall, the uptake and release of resazurin in protoplasts may be impacted [22].

In this study, we used resazurin to develop a rapid, sensitive, and high throughput assay to measure the metabolic activity of protoplasts. We also demonstrate that this assay effectively measures the cytotoxicity of a range of nanomaterials.

## Materials and methods

### Protoplast processing

#### Seeds germination

Seeds of canola (*Brassica napus*, L.; cultivar Westar) were surface sterilized with 70% ethanol for 1 min, followed by 3–4 rinses in sterile double-distilled water and then 20% w/v commercial bleach (containing 5% sodium hypochlorite) and 0.1% w/v Tween-20 for 30 min with continuous stirring. Then, they were rinsed 3–4 times with sterile water. Germination of the surface-sterilized 100 seeds is carried out under controlled conditions (22 °C; darkness) in glass jar vessels containing 40 ml of Murashige and Skoog (MS) basal medium (4.33 g/L; Sigma-Aldrich, USA), pH 5.8, solidified with bacto-agar (7 g/L; Sigma-Aldrich, Mexico).

#### Protoplast isolation

**Fig. 1:**
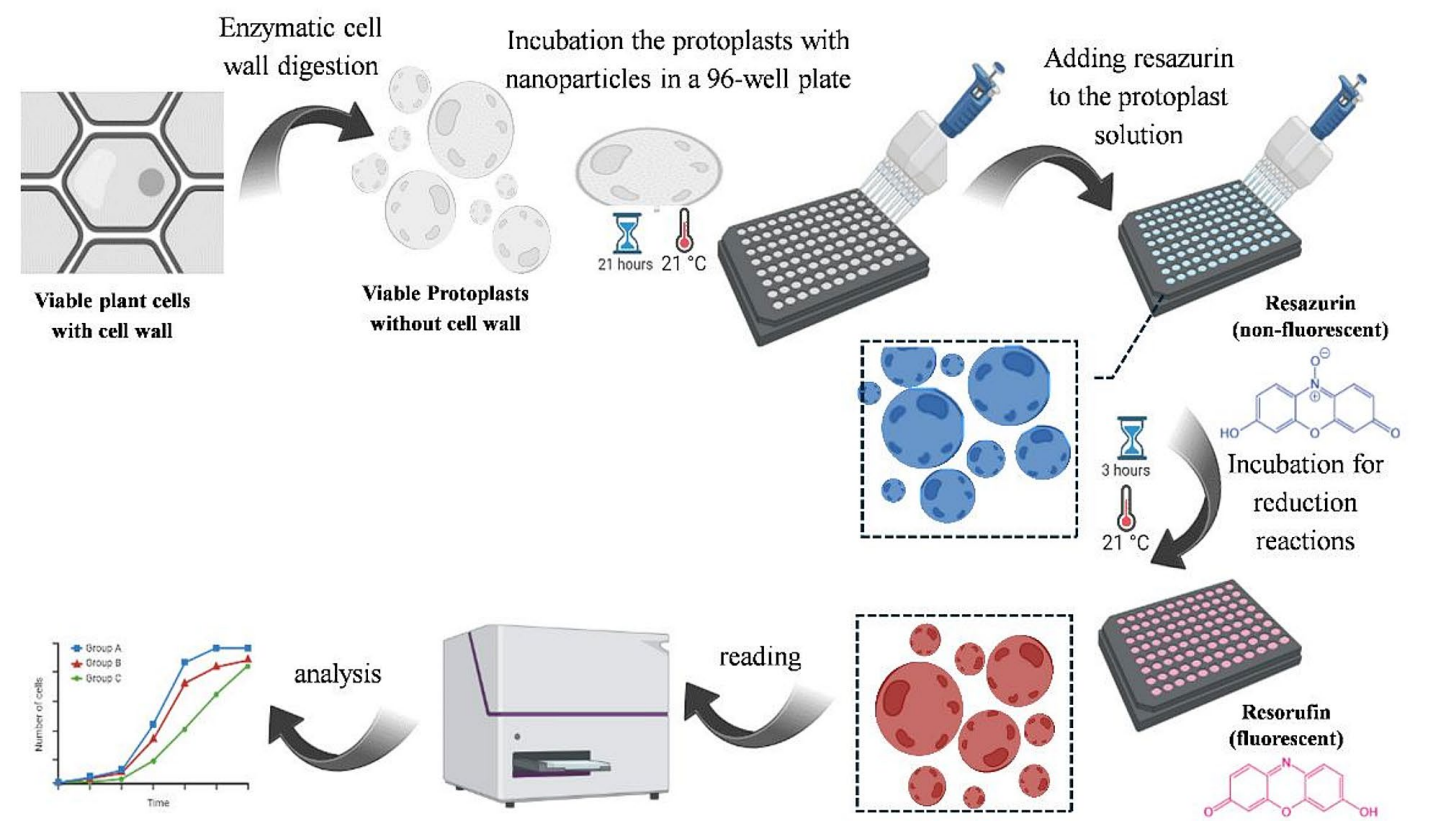
Workflow of the resazurin assay to determine the nanoparticle effects on canola protoplast viability. This figure was generated with www.BioRender.com

In our study, the method involved several key steps (Fig. 1). First, protoplasts were isolated from hypocotyl canola. Protoplast isolation from young canola seedlings’ hypocotyls was conducted based on protocols established by Yoo and Li [30] with some modifications. About 80 young hypocotyl segments, 5 mm in length, from 5–7-day-old seedlings were sliced into fine pieces on 1–2 drops of sterile water in a sterile Petri dish and incubated in plasmolysis solution (0.3 M sorbitol and 0.05 M CaCl_2_ at pH 5.7) for 1 h at room temperature in the dark, without shaking. The hypocotyl pieces were treated with 10 mL of enzyme solution and incubated for 14–16 h at room temperature in the dark with gentle shaking. To prepare the enzyme solution, 1.5% (w/v) cellulase (Onozuka R-10, RPI Corp., USA) and 0.6% (w/v) Macerozyme (R-10, RPI Corp., USA) were mixed with 0.4 M mannitol and 10 mM MES (2-Morpholinoethanesulphonic acid). The mixture was heated to 55 ˚C for 10 min in a water bath to deactivate the proteases, then cooled to room temperature. After cooling, 0.1% (w/v) bovine serum albumin (BSA), 1 mM CaCl_2_, and 1 mM β-mercaptoethanol were added, and the solution was adjusted to pH 5.7 and mixed gently. The prepared enzyme solution was sterilized using a 0.22 μm filter, then aliquoted in a laminar flow hood and stored at -20 ˚C. Before use, the solution was thawed on ice and mixed thoroughly.

The protoplast-containing solution was first filtered through a 150 μm nylon mesh to remove large particles and then through a 70 μm nylon cell strainer to eliminate smaller debris and undigested cell walls. The filtered protoplast suspension was diluted with 30 ml of W5 salt solution (154 mM NaC1, 125 mM CaC1_2_, 5 mM KC1, 2 mM MES; pH = 5.7). Protoplasts were gently pelleted by centrifugation for ten minutes at 100 x g and the supernatant was removed. Then, 10 ml of W5 solution was slowly added to the re-suspended protoplasts, followed by centrifugation at 100 x g for 5 min, and this process was repeated twice to ensure the removal of debris and excess enzymes. Pellets were then re-suspended in 5 ml precooled W5 solution and incubated on ice in the dark for 1 h. The supernatant was discarded, and the protoplasts were carefully re-suspended in the W5 solution. If cell debris was detected, this step was repeated. As mentioned, for protoplast isolation, pellets were re-suspended in 5 ml of precooled W5 solution to achieve the appropriate concentration for counting. In subsequent experiments involving the treatment of protoplasts with resazurin, either W5 or MMG buffer (0.4 M mannitol, 15 mM MgCl_2_, 4 mM MES; pH = 5.7) was used.

The protoplast solution was mixed with 10 µl trypan blue and loaded on a hemocytometer for counting under a light microscope. The percentage of viable cells was calculated as follows:

$$Total\begin{array}{*{5}{c}}{}\end{array}cells/ml{\rm{ }} = {\rm{ }}Total\begin{array}{*{5}{c}}{}\end{array}cells\begin{array}{*{5}{c}}{}\end{array}counted \times \frac{{dilution\begin{array}{*{5}{c}}{}\end{array}factor}}{{\# of\begin{array}{*{5}{c}}{}\end{array}squares}} \times 10000$$

$$\begin{array}{l}Viable\begin{array}{*{20}{c}}{}\end{array}cells\begin{array}{*{20}{c}}{}\end{array}\left( \% \right){\rm{ }} = \\\frac{{Total\begin{array}{*{20}{c}}{}\end{array}number\begin{array}{*{20}{c}}{}\end{array}of\begin{array}{*{20}{c}}{}\end{array}viable\begin{array}{*{20}{c}}{}\end{array}cells\begin{array}{*{20}{c}}{}\end{array}\left( {Unstained} \right)}}{{Total\begin{array}{*{20}{c}}{}\end{array}number\begin{array}{*{20}{c}}{}\end{array}of\begin{array}{*{20}{c}}{}\end{array}viable\begin{array}{*{20}{c}}{}\end{array}\left( {Unstained} \right)\begin{array}{*{20}{c}}{}\end{array}and\begin{array}{*{20}{c}}{}\end{array}nonviable\begin{array}{*{20}{c}}{}\end{array}\left( {stained} \right)\begin{array}{*{20}{c}}{}\end{array}cells}} \times {\rm{ }}100\end{array}$$

When protoplasts were counted using trypan blue and a hemocytometer, it was observed that the trypan blue dye formed aggregates when the protoplasts were resuspended in either W5 or MMG buffer (Supplementary file S1). This phenomenon is likely due to Ca²⁺ ions in the W5 buffer and Mg²⁺ ions in the MMG buffer, which bind to the sulfonate groups of the trypan blue dye, thereby reducing its solubility. Therefore, W5 and MMG buffer without CaCl_₂_ and MgCl_₂_ was used whenever protoplasts were dyed with trypan blue and counted on a hemocytometer.

### Viability assays

#### Buffer preparation

We used three common buffers to test their effects on resazurin reduction: W5, W5-MES (154 mM NaCl, 125 mM CaCl₂, 5 mM KCl, 2 mM MES, pH 5.7), and MMG. These buffers were prepared on the same day of use as assay medium/buffer. The impact of sugars on resazurin was assessed by varying glucose concentrations (2.5, 5, and 10 mM) in W5 buffer and mannitol concentrations (0.2, 0.4, and 0.8 M) in MMG buffer.

#### Optimization of resazurin assay in the absence of protoplasts

To determine the optimal concentration of resazurin, resazurin dye solutions (#R7017, Sigma-Aldrich, St. Louis, MO, USA) were prepared at concentrations of 4, 20, 40, 60, and 80 µM. The mixture of buffers and resazurin dye solution in a final volume of 200 µl was transferred to a 96-well plate (black plate with clear bottom, Thermo Scientific™, Canada) and incubated at room tempreture in the dark. Then, the samples were analyzed either spectrophotometrically by measuring the absorbance at a wavelength of 600 nm or fluorometrically by measuring fluorescence at a wavelength of 590 nm using an excitation wavelength of 540 nm (Microplate Reader, Varioskan^™^ LUX, Vantaa, Finland). Resazurin stability was assessed by incubating resazurin in the buffers for up to 5 h. Readings were obtained every consecutive hour for a total of 5 h. A control was included to measure any potential effect of the buffer alone on resazurin fluorescence in the absence of protoplasts. Additionally, the impact of heating on resazurin reduction was assessed by heating buffers to 90 °C for 20 min. To evaluate the effects of solvents and surfactants, buffers containing 20% (v/v) dimethyl sulfoxide (DMSO) and 0.1% (v/v) Triton X-100 were tested.

#### Optimization of resazurin assay in the presence of protoplasts

To determine the protoplast density for sufficient resazurin intensity within a reasonably short incubation period, protoplasts with different densities (5,000; 10,000; 15,000; 20,000; 25,000; 30,000; 35,000; 40,000; 45,000; 50,000; 55,000; and 60,000) were incubated with 40 µM resazurin at various temperatures (4, 10, 20, and 30 ˚C) over 24 h incubation period. The fluorescence and absorbance were read under the same conditions. To optimize the resazurin assay for samples containing protoplasts, various controls were employed. Untreated protoplasts and protoplasts treated with 20% DMSO served as controls. Additionally, a sample was included to measure the metabolic activity of protoplasts without resazurin to assess the autofluorescence of the protoplasts, which refers to the natural fluorescence emitted by certain compounds within them.

### Nanomaterials processing

#### Synthesis and preparation of nanomaterials

Commercial silica nanosphere (SiO_2_ NS) of 20, 50, and 100 nm and silver nanospheres (Ag NS) of 20, 50, and 100 nm were purchased from nanoCompoxis (San Diego, CA, USA). Commercial SiO_2_ NS of ca. 12 nm sizes were purchased from Sigma Aldrich. The lab-synthesized SiO_2_ NS include 40 and 230 nm sizes. These particles were synthesized by the modified Stöber method [31]. For the synthesis of 40 nm SiO_2_ NS, anhydrous methanol (200 ml), Milli-Q water (54.2 ml), and ammonium hydroxide (28–30%, 12.5 ml) were mixed by stirring at 1000 rpm in a 500 ml round bottom flask at room temperature for 10 min. Then, 12.5 ml of tetraethoxysilane (TEOS 98%) was added in a single step and stirred for 1 h. Afterward, the SiO_2_ NS were isolated by centrifugation at 10,000 rpm for 30 min. Then, the precipitate was resuspended in 80 ml reagent alcohol and precipitated by centrifugation at 4,400 rpm for 30 min (repeated three times). Finally, the precipitate was resuspended in 30 ml Milli-Q water and lyophilized for 72 h, yielding 4.3 g SiO_2_ NS. To synthesize 230 nm SiO_2_ NS, 732 ml 100% Ethanol, 103 ml Milli-Q water, and 60 ml ammonium hydroxide (28–30%) were mixed by stirring at 1000 rpm at 50 $\:^\circ\:$C for 1 h. Then, 105 ml TEOS (98%) was added in one shot and stirred for 2 h at 1000 rpm at 50 $\:^\circ\:$C. SiO_2_ NS were isolated by centrifugation at 1000 rpm for 10 min. The precipitate was resuspended in 100 ml reagent alcohol and precipitated by centrifugation at 4,400 rpm for 30 min (repeated three times). Finally, the precipitate was resuspended in 200 ml Milli-Q water and lyophilized for 96 h, yielding 30 g SiO_2_ NS. A stock solution of each SiO_2_ NS (5 mg/ml) was prepared by dissolving 25 mg SiO_2_ NS in Milli-Q water by stirring for 10 min and sonication for 10 min.

Lipid nanoparticle (LNP) were made with 2-dioleyloxy-N, N-dimethyl-3-aminopropane (DODMA) and pC1302-GFP-M05 DNA plasmids (a gifted from the University of Saskatchewan) based on a published protocol [32] with some modifications. Briefly, LNPs were prepared by combining appropriate volumes of cationic lipid (DODMA: 50 mol%), helper lipid (DOPE; 11.5 mol%), PEGylated lipid (PEG-DMPE; 1.0 mol%), and cholesterol (37.5 mol%) in anhydrous ethanol with plasmid DNA in 25 mM sodium acetate (pH 4.0). DNA concentration in the aqueous phase was 0.133 mg/ml, with a target molar charge ratio (mol cationic lipid: mol DNA, N/P) of 4:1. The preparation was carried out using a NanoAssemblr Benchtop instrument and microfluidic cartridges (Precision Nanosystems, Vancouver, BC, Canada) set to a combined flow rate of 9 ml/min [flow rate ratio of 3 (DNA/ aqueous) to 1 (ethanol/lipid)]. The total lipid concentration was maintained at 13.70 mM. DNA encapsulation efficiencies were measured using a Quant-iT PicoGreen dsDNA Assay Kit (ThermoFisher Scientific).

The cholesteryl butyrate nanoemulsion (Chol-but NE) was prepared as described previously [33] with some modifications. Briefly, a warm oil-in-water microemulsion was prepared from cholesteryl butyrate (Sigma Aldrich, C4758) (12%), Epikuron 200 (Cargill, Milan, Italy) (15%), sodium taurocholate (Sigma Aldrich, 86339) (3%) and water. Butanol (Sigma–Aldrich) (11%) was added as a preservative. The warm microemulsion was dispersed (1:10, v/v) in cold water and the resulting cholesteryl butyrate aqueous dispersion was filtered through fiberglass. The nanoemulsion was washed using a float-a-lyzer.

#### Characterization of nanomaterials

Characterization of the nanoparticles, including SiO₂ NS, Ag NS, LNP, and Chol-but NE, involved several analytical techniques. The Z-average diameter and size distribution of the nanoparticles were determined using Dynamic Light Scattering (DLS) with a Zetasizer Nano ZS (Malvern Instruments, Malvern, Worcs, UK). To assess the size, shape, and uniformity of the nanoparticles, as well as to perform detailed surface characterization, Scanning Electron Microscopy (SEM) was employed. Light microscopy was used to visually assess the impact of nanoparticles on cell structure and integrity.

#### Treatment of protoplasts with nanomaterials

To validate our optimized resazurin assay, the isolated protoplasts were treated with various types of nanoparticles (Fig. 1) to study their effects on protoplast viability and stress responses. These nanomaterials included citrate-capped silver nanospheres (Ag NS) (in three sizes: 20, 50, and 100 nm); SiO_2_ NS standard (in three sizes: 20, 50, and 100 nm); and other commercial and lab synthesized SiO_2_ NS (in three sizes: 12, 40, and 230 nm) and Chol-but NE, and LNP (172.5 nm). The nanoparticles were used at 5, 50, and 500 ng/ml. In each sample, 10 µl of 40 µM resazurin was added to 20 µl of nanoparticles (at the aforementioned concentrations) and 70 µl of protoplast solution (containing approximately 20,000 protoplasts). Samples without nanoparticles or with 20% DMSO served as controls. The samples containing protoplasts were incubated with nanoparticles at room temperature for 21 h, after which resazurin was added, and the incubation continued for an additional 3 h until a change in resazurin color was observed. Readings of the signals were obtained either spectrophotometrically or fluorometrically (Fig. 1). Three independent experiments were conducted. As described above, fluorescence was measured at an excitation wavelength of 540 nm and an emission wavelength of 590 nm, or absorbance was measured at 600 nm with a reference wavelength of 690 nm. The absorbance at the reference wavelength of 690 nm was subtracted from the absorbance measured at 600 nm.

### Statistical analysis

Statistical analyses and graph plotting were conducted using GraphPad Prism 10 (GraphPad Software, LLC, version 10.2.2). Data were expressed as mean ± standard deviation (SD). Statistical significance was evaluated using one-way ANOVA followed by Dunnett’s test (*p* < 0.05).

## Results

### Optimizing buffers and resazurin concentration

**Fig. 2:**
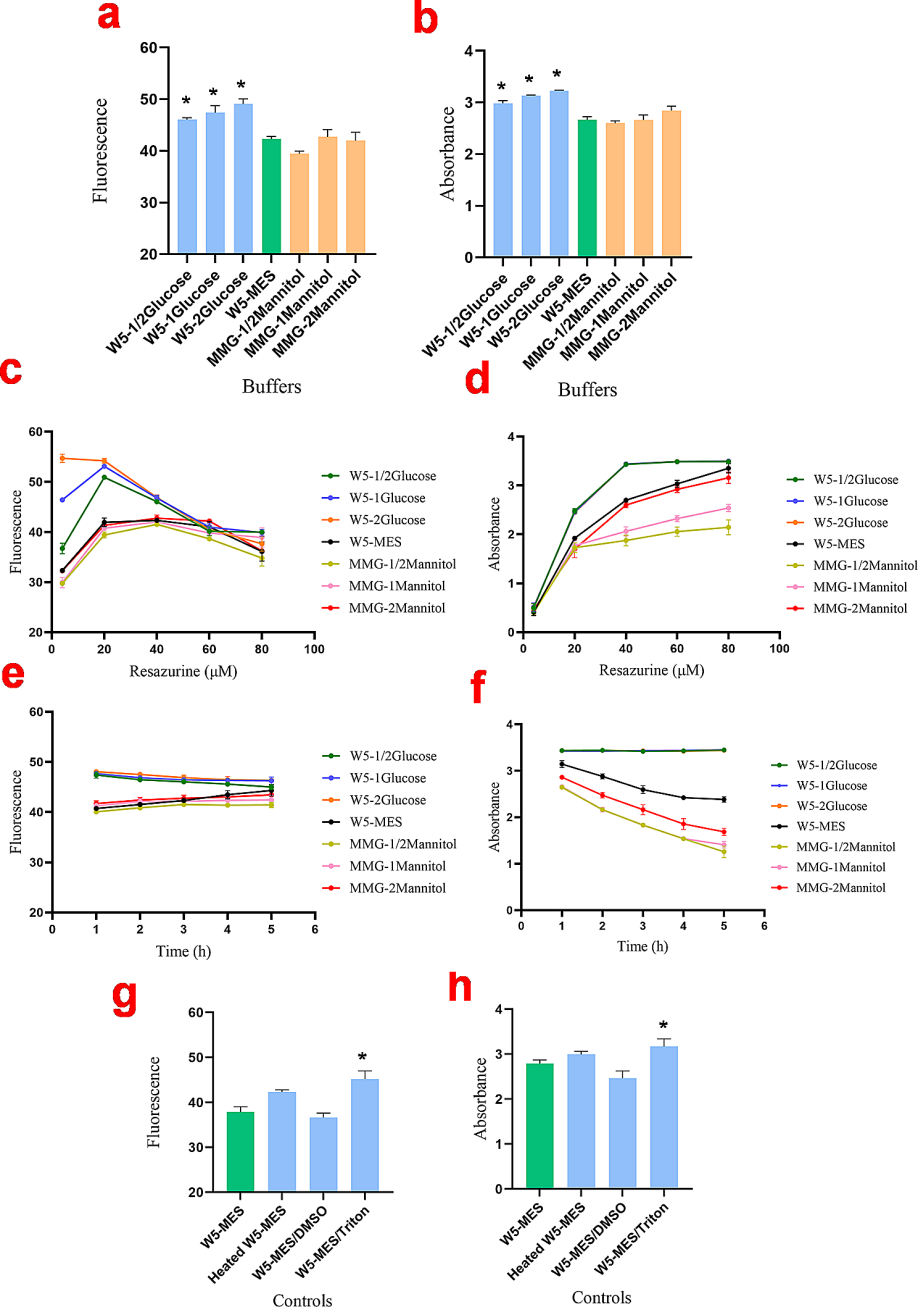
Optimization of resazurin concentration, buffer types, and incubation time for the resazurin assay in the absence of the protoplasts. **a** and **b**: Fluorescence intensity and absorbance values of different buffers including, W5 buffer with ½ Glucose (2.5 mM), 1 Glucose (5 mM), and 2 Glucose (10 mM), W5 buffer with MES (2 mM), MMG solution with ½ mannitol (0.2 M), 1 mannitol (0.4 M), and 2 mannitol (0.8 M) after 3 h incubation with 40 µM resazurin. **c** and **d**: Fluorescence intensity and absorbance values of different buffers after 3 h incubation with various concentrations of resazurin (4, 20, 40, 60, and 80 µM). **e** and **f**: Fluorescence intensity and absorbance values of different buffers at different time intervals. **g** and **h**: Fluorescence intensity and absorbance values of various types of controls. For the heated W5-MES samples, buffers were heated at 90 °C for 20 min. The dimethyl sulfoxide (DMSO) and Triton X-100 were used at concentrations of 20% (v/v) and 0.1% (v/v), respectively. Results are representative of three independent experiments. Error bars represent mean ± Standard Deviation (STD). *Significantly reduced compared to control (*P* < 0.05), as calculated by one-way ANOVA with Dunnet’s t-test

We assessed whether protoplast buffers containing various sugars could reduce resazurin in the absence of cells. The fluorescence data revealed that glucose in the W5 buffer increased the fluorescence of resazurin compared to the W5 + MES buffer, which lacks sugar (Fig. 2a and b). Conversely, the MMG buffer containing mannitol did not significantly affect resazurin fluorescence. Increasing concentrations of mannitol did not lead to significant reductions in resazurin fluorescence (Fig. 2c and d). The optimal concentration of resazurin in the W5 buffer containing glucose was 20 µM. Resazurin concentrations of 20 or 40 µM were tested in the W5 + MES and MMG buffers.

Based on the experimental data, 40 µM resazurin in W5 + MES buffer was chosen for subsequent analyses (Fig. 2c and d). Figure 2e and f illustrate the stability of resazurin over a 5-hour incubation with the W5 buffer. The data indicate that resazurin fluorescence and absorbance remained relatively stable across the different buffers tested.

Figure 2g and h show the effect of DMSO and Triton in the W5 + MES buffer on resazurin fluorescence. Interestingly, Triton increased resazurin fluorescence suggesting reduction. DMSO did not change resazurin fluorescence. It has been suggested that autoclaving resazurin can cause it to be reduced [22]. However, autoclaving the samples did not significantly affect resazurin levels (Fig. 2g and h). Therefore, DMSO (20%) was selected as the appropriate solvent for subsequent analyses.

### Optimizing protoplast number, temperature, and time of incubation

**Fig. 3:**
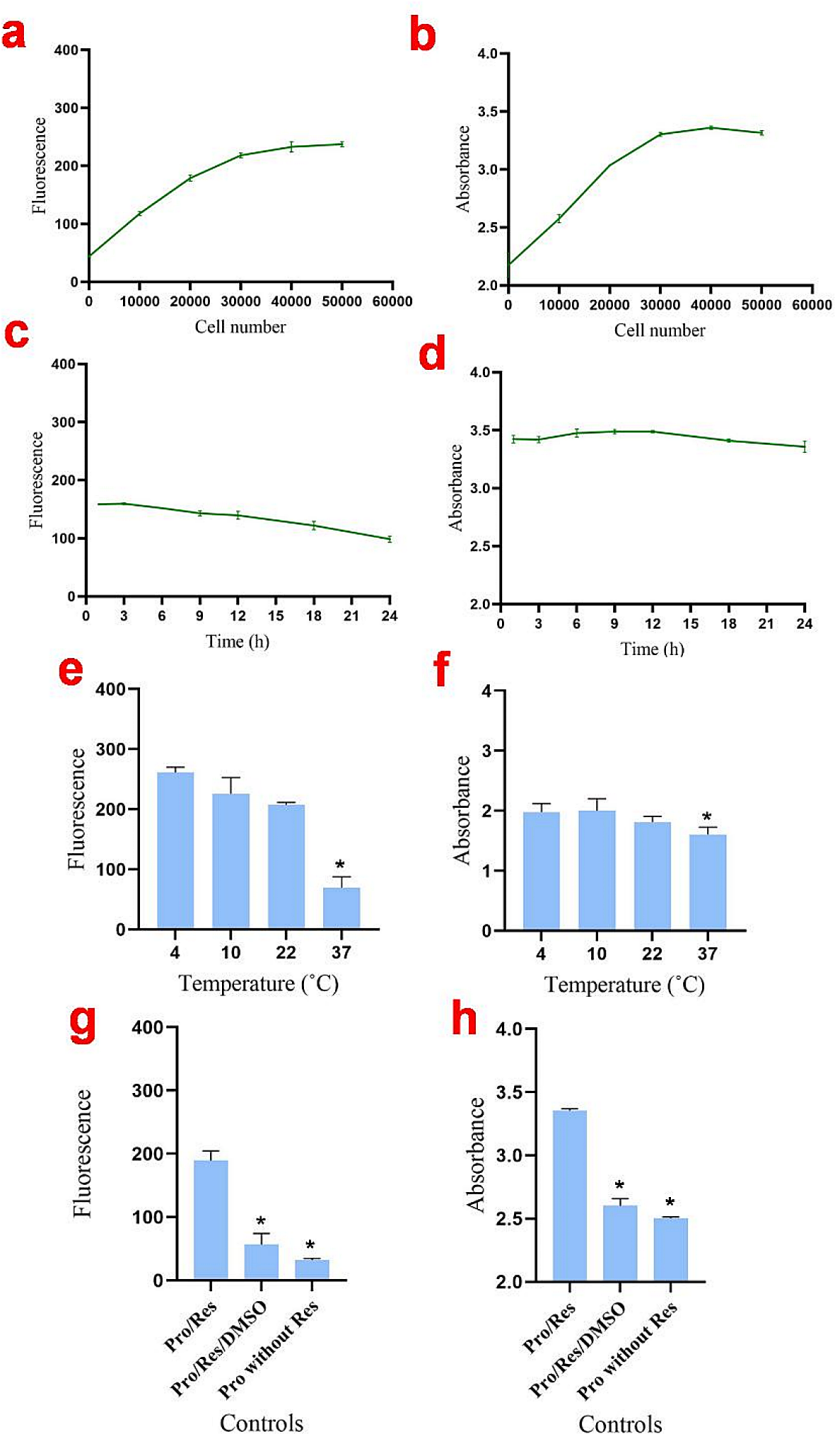
Optimization of protoplast number, incubation time, and temperature for resazurin assay using canola hypocotyl protoplasts. **a** and **b**: Fluorescence intensity and absorbance values of different protoplasts density after 3 h incubation with 40 µM resazurin. **c** and **d**: Fluorescence intensity and absorbance values of approximately 20,000 protoplasts per well at various time points. **e** and **f**: Fluorescence intensity and absorbance values of 20,000 protoplasts per well after 24 h of incubation with 40 µM resazurin at different temperatures. **g** and **h**: Fluorescence intensity and absorbance values of various types of controls after 3 h incubation with 40 µM resazurin. Pro: protoplast, Res: resazurin. The dimethyl sulfoxide (DMSO) was used at a concentration of 20% (v/v). Results are representative of three independent experiments. Error bars represent mean ± Standard Deviation (STD). *Significantly reduced compared to control (*P* < 0.05), as calculated by one-way ANOVA with Dunnet’s t-test

The size of isolated hypocotyl protoplasts was 38 ± 13 μm. Both the absorbance and fluorescence measurements demonstrated an initial increase followed by a plateau in the relationship between cell number and the reduction of resazurin (Fig. 3a and b). When more than 30,000 protoplasts per well were employed, a slight decrease in resazurin intensity was noted (see Fig. 3a and b). A protoplast density of approximately 20,000 cells per well gave the most significant fluorescence intensity within 3 h (Fig. 3a-d). Extending the incubation to 24 h resulted in a decreased fluorescence intensity compared to 3 h (Fig. 3c and d). The results indicated that resazurin likely does not have significant toxic effects on protoplasts, as evidenced by the approximately linear response observed over 24 h of incubation (Fig. 3c and d).

Among the various incubation periods examined, a 3-hour incubation period showed the least reduction in fluorescence intensity (refer to Fig. 3c and d). As a result, a cell density of approximately 20,000 cells per well and a 3-hour incubation time were chosen as the optimal assay conditions for subsequent analyses.

The results showed that protoplasts were the most metabolically active at 4 to 22 °C (Fig. 3e and f). However, higher temperatures (e.g., 37 °C) caused decreased metabolic activity of the protoplasts. Consequently, we selected 4 °C as the storage temperature and 22 °C as the incubation temperature for the protoplasts in the resazurin assay conducted in this study. The evaluation of autofluorescence intensity of the protoplasts in the absence of resazurin revealed that protoplasts did not exhibit autofluorescence at 590 nm (Fig. 3g and h).

**Fig. 4:**
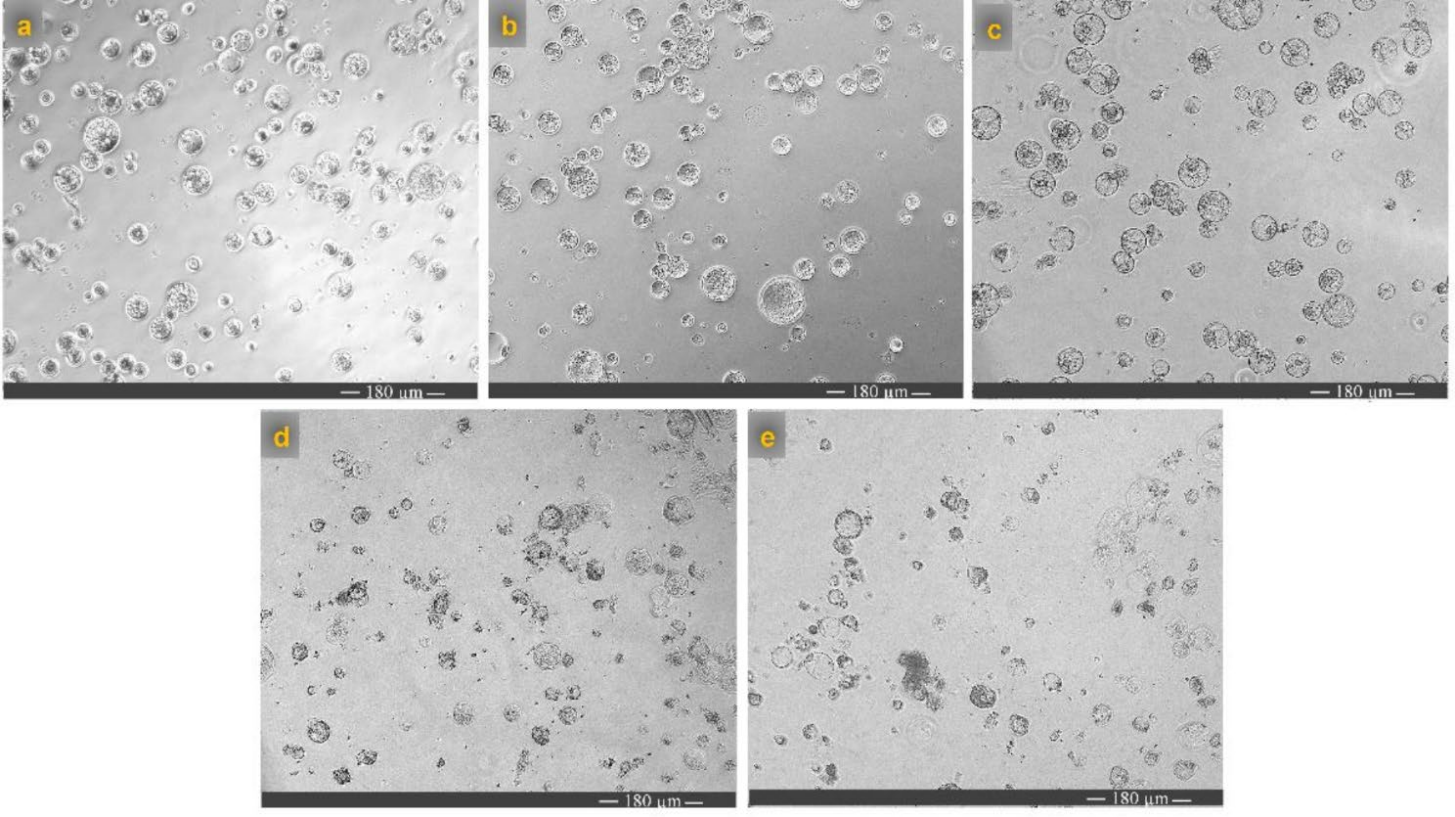
Light microscopy images (10×) of canola hypocotyl protoplasts incubated for 24 h at various temperatures. (**a**) 4 ˚C, (**b**) 10 ˚C, (**c**) 22 ˚C, (**d**) 37 ˚C. (**e**) The protoplast was treated with 20% DMSO. W5 buffer containing MES was used as the assay buffer for all samples

Figure 4 illustrates the morphological changes in canola protoplasts over a 24-hour incubation period at different temperatures. The findings revealed that DMSO (20%) significantly decreased the fluorescence intensity in the presence of protoplasts, making it a suitable control for this assay (Fig. 3g and h, and Fig. 4).

### Characterization of the nanomaterials used to validate the assay

**Fig. 5:**
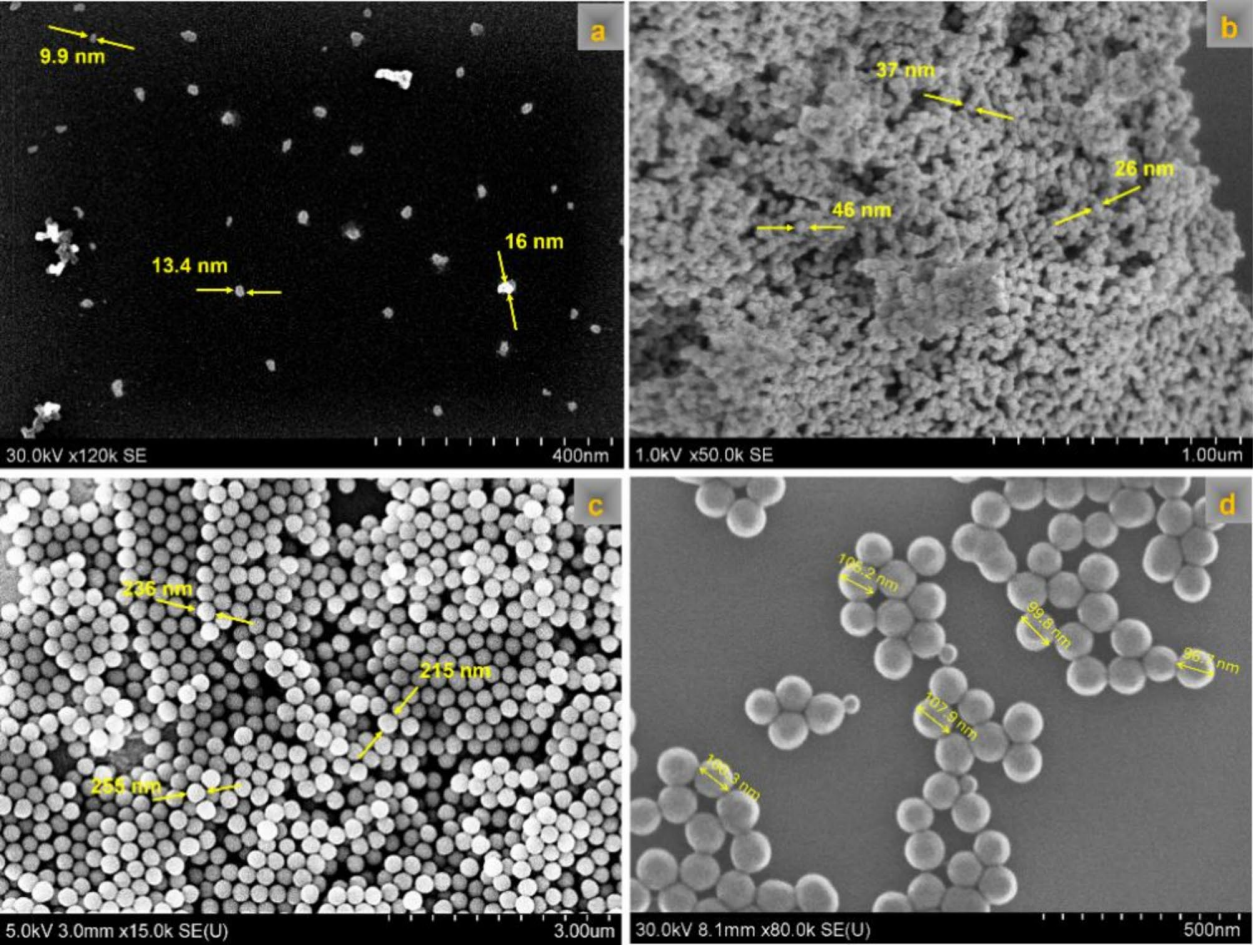
Scanning electron microscopy (SEM) images and particle size distributions of SiO_2_ and Ag NS. (**a**) commercial 12 nm SiO_2_ NS, (**b**) lab-synthesized 40 nm SiO_2_ NS standard, (**c**) lab-synthesized 230 nm SiO_2_ NS standard, and (**d**) 100 nm SiO_2_ NS standard

The SEM images of the commercial (Fig. 5a) lab-synthesized SiO_2_ NS (Fig. 5b and c) and the SiO_2_ NS standard (Fig. 5d) indicated that these nanospheres were of the expected size and shape. The results also showed that the particles have a uniform size and shape, indicating minimal variability.

The zeta potential measurements for citrate-capped Ag NS and SiO_2_ NS, each in different sizes, are summarized in Table 1 and Supplementary file S2. The zeta potential measurements indicated that all nanoparticles possess a negative surface charge (Table 1).

**Table 1:** Physicochemical properties of nanoparticles used in toxicity studies

Nanoparticle type	Size measured by SEM (nm)	Size measured by DLS (nm)	Zeta Potential (mV)
Ag NS; citrate	20 ± 3^*^	13.5 ± 2.69	-43 ± 12.49
	50 ± 4^*^	78.8 ± 3.38	-45.9 ± 9.15
	100 ± 8^*^	106 ± 6.15	-55.4 ± 9.91
SiO_2_ NS; standard	20 ± 4^*^	32.7 ± 2.21	-30.2 ± 9.15
	50 ± 4^*^	50.7 ± 10.34	-45.8 ± 7.34
	100 ± 4^*^	114 ± 1.17	-26.9 ± 5.46
Lab-synthesized SiO_2_ NS	12 ± 3.7	-	-48.1 ± 6.56
	40 ± 5.8	-	-36.5 ± 4.64
	230 ± 19.5	-	-56.9 ± 6.38

Each sample was measured in triplicate, and data are presented as the mean ± standard deviation. SEM: Scanning Electron Microscopy. *The particle size and STD were determined by nanoCompoxis supplier

### Measuring the effect of nanomaterials on protoplast viability

**Fig. 6:**
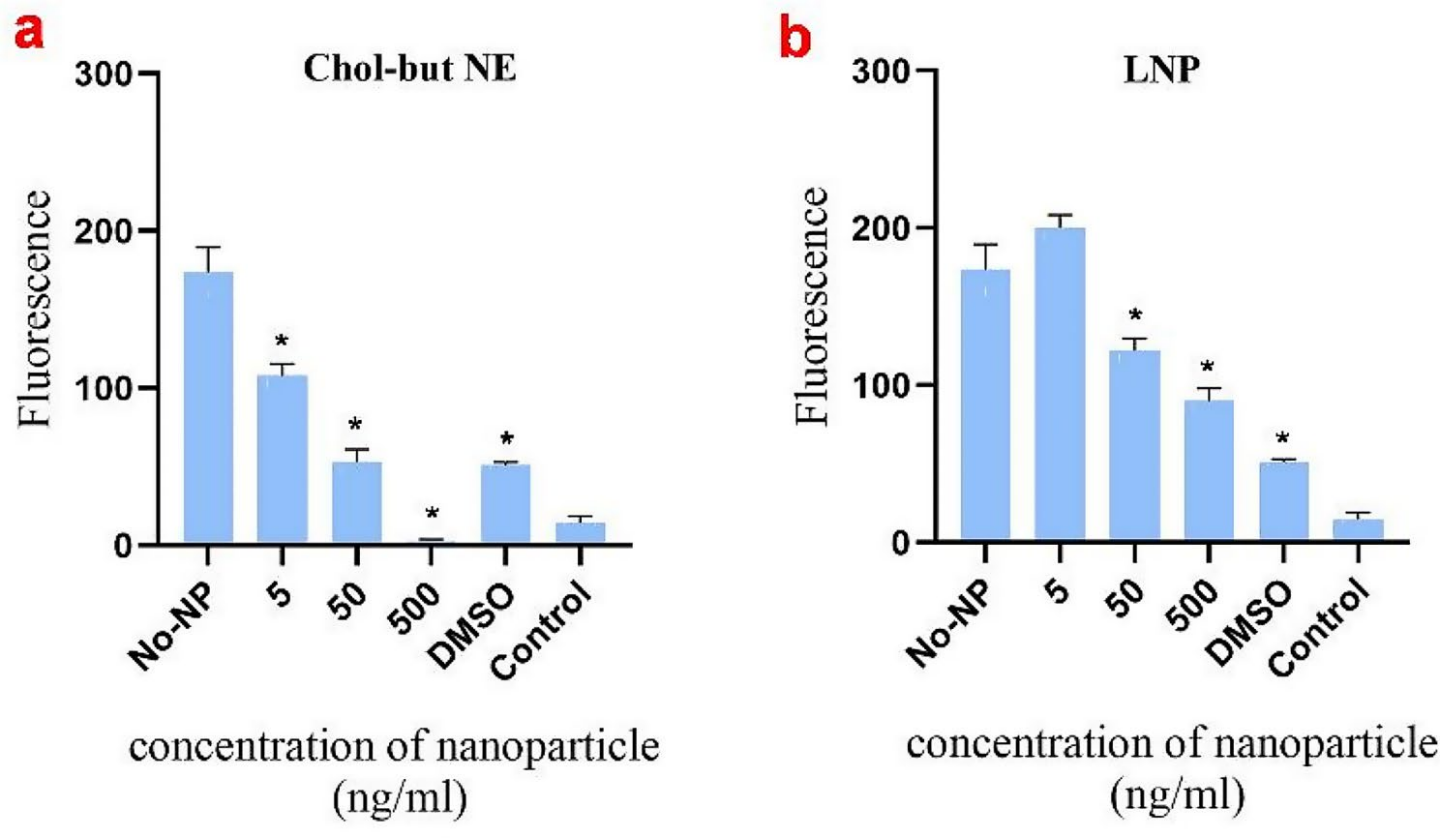
Resazurin assay on canola protoplasts after 24 h of exposure to nanomaterials at various concentrations. (**a**) cholesterol + butyrate nanoemulsion (Chol-but NE) and (**b**) lipid nanoparticles (LNP) (172.5 nm). Protoplasts solution (containing approximately 20,000 protoplasts) was treated with different concentrations of nanoparticles (5, 50, and 500 ng/ml) for 21 h, followed by the addition of 40 µM resazurin for the remaining 3 h. The samples that were served as controls include No-NP, W5-MES + 40 µM resazurin + protoplasts mixture; DMSO, W5-MES + 40 µM resazurin + protoplasts + 20% DMSO; and Control, nanoparticle only (no resazurin). Results are representative of three independent experiments. Error bars represent mean ± Standard Deviation (STD). *Significantly reduced compared to control (*P* < 0.05), as calculated by one-way ANOVA with Dunnet’s t-test

**Fig. 7:**
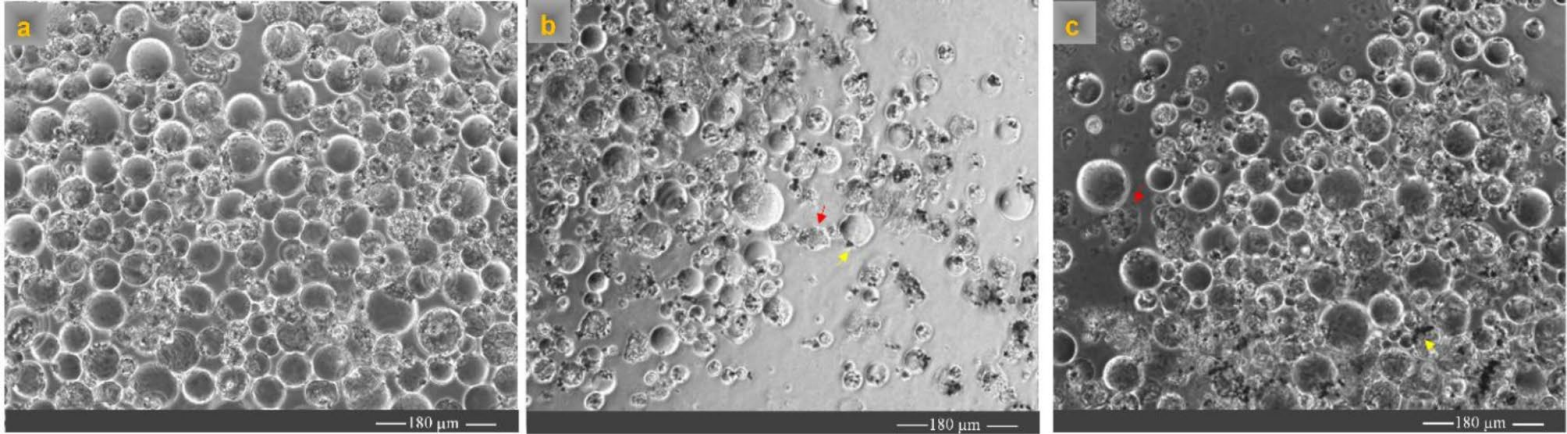
Light microscopy (10× magnification) analyses of protoplasts 24 h after treatment with various nanoparticles. The bright field images of (**a**) the non-treatment canola protoplasts and treatment with (**b**) the cholesteryl-butyrate nanoemulsion (Chol-but NE) and (**c**) lipid nanoparticles (LNP) at 500 ng/ml. The yellow arrows indicate nanoparticles, while the red arrows highlight dead protoplasts

The impact of nanomaterials on protoplast metabolic activity showed that Chol-but NE and LNP decreased protoplast metabolic activity in a concentration-dependent manner (Fig. 6a and b). Both absorbance and fluorescence measurements exhibited a significant decrease in metabolic activity after 24 h of exposure to the Chol-but NE and LNP. Light microscopy analyses of the protoplasts treated with the Chol-but NE and LNP confirmed the presence of dead cells (Fig. 7b and c).

**Fig. 8:**
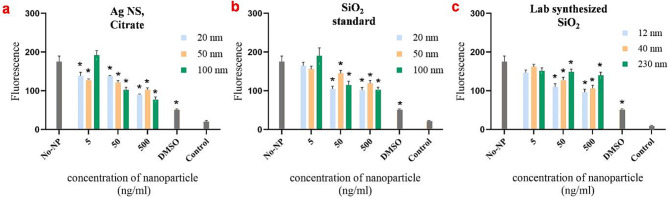
Resazurin assay on canola protoplasts after 24 h of exposure to various nanoparticles. (**a**) silver nanospheres (Ag NS); citrate (in three sizes: 20, 50, and 100 nm); (**b**) silica nanospheres (SiO_2_ NS); standard (in three sizes: 20, 50, and 100 nm); and (**c**) lab-synthesized SiO_2_ (at sizes of 12, 40, and 230 nm). Protoplasts solution (containing approximately 20,000 protoplasts) was treated with nanoparticles at concentrations of 5, 50, and 500 ng/ml for 21 h, followed by the addition of 40 µM resazurin for the remaining 3 h. The samples that served as controls include No-NP, W5-MES + 40 µM resazurin + protoplasts mixture; DMSO, W5-MES + 40 µM resazurin + protoplasts + 20% DMSO; and Control, nanoparticle only (no resazurin). Results are representative of three independent experiments. Error bars represent mean ± Standard Deviation (STD). *Significantly reduced compared to control (*P* < 0.05), as calculated by one-way ANOVA with Dunnet’s t-test

The results showed that both the SiO_2_ NS standard and the Ag NS decreased the metabolic activity of the protoplasts at concentrations above 5 ng/ml (Fig. 8). The size of the Ag NS did not show a clear effect on metabolic activity. However, the largest Ag NS (100 nm) exhibited increased cytotoxicity at 50 and 500 ng/ml concentrations. The other commercial and lab-synthesized SiO₂ NS also showed size and concentration-dependent cytotoxicity, though less than that of Ag NS and the standard SiO₂ NS. In our study, the fluorescence of the nanoparticles was also measured (Figs. 6 and 8). None of the nanomaterials were fluorescent at 590 nm.

## Discussion

Protoplasts are extensively utilized in plant science research and crop breeding, particularly for transfection, because the absence of the cell wall and cuticle facilitates macromolecules delivery, including nanoparticles [34, 35]. Our isolated hypocotyl protoplasts were consistent in size with previous reports [36]. Although nanomaterials are frequently used to modify plant cells and tissues, their effect on cytotoxicity is under-explored. A significant challenge is the lack of rapid, reproducible biochemical analysis of plant cell metabolism. We aimed to develop an assay to measure plant cell metabolism using the dye resazurin (also called Alamar blue), which is reduced by NADH, NADPH, FADH, and other reductive species to resorufin [37, 38]. In addition to the shift in absorbance from 600 to 570 nm, the excitation of resorufin at 570 nm can cause fluorescence emission at 590 nm [39]. These characteristics can be used to design a rapid and sensitive assay of cellular metabolism. Our finding aligns with previous research by Byth and colleagues that applied resazurin-based assays to plant cell viability [22]. This study optimized the resazurin assay for tomato cell suspension cultures and compared it with the conventional 2,3,5-triphenyltetrazolium chloride (TTC) assay. They demonstrated that the resazurin assay exhibited a sigmoidal relationship between cell viability and resazurin reduction, like the TTC assay, and effectively detected significant decreases in cell viability after pathogen exposure. Notably, the resazurin assay proved to be more rapid, versatile, and non-toxic than the TTC assay, which also enabled subsequent cell analysis.

Due to its chemical structure, resazurin can be reduced by sugars and other buffer components. Our analysis revealed that glucose in the W5 buffer reduces resazurin in the absence of protoplasts, whereas mannitol in the MMG buffer does not. This indicates that fluorescence increase is linked to glucose concentration in the W5 buffer. Reducing sugars, like glucose, can interfere with the resazurin assay for plant cell viability, causing false positives. In contrast, non-reducing sugars such as mannitol and sucrose are less likely to affect the assay because the glycosidic linkage of sucrose prevents it from directly participating in oxidation-reduction reactions with resazurin. Similarly, mannitol, a sugar alcohol, lacks a free aldehyde or ketone group necessary for oxidation-reduction reactions. Byth et al. found that MS and Hoagland’s media reduced resazurin without cells present but did not specify the reducing capacities of other components [22]. We employed the W5 + MES buffer for our assays due to its minimal impact on resazurin reduction and lack of reducing sugars. This protein-free buffer maintains osmotic balance and supports precise evaluation of plant cell metabolic activity without external interference.

Our results showed that the fluorescence intensity of resazurin remained largely stable over a 5-hour incubation period in the W5 buffer, indicating that resazurin was neither naturally degraded nor significantly affected by the buffer. This stability is crucial as it confirms that the buffers used did not interfere with the resazurin’s reliability as an indicator. Consequently, this supports the validity of resazurin as a consistent measure of metabolic activity in our assays. It is essential to consider this parameter to accurately interpret assay results and ensure that any changes in resazurin fluorescence are attributable to the metabolic activity of the cells rather than non-specific reductions of resazurin over time.

In our study, we found that the observed optimal concentration of resazurin for the W5 buffer containing glucose was 20 µM, but for W5 + MES and MMG buffers, either 20 or 40 µM could be used. However, increasing the percentage of resazurin above 55 µM is not recommended due to the potential interference with cellular processes and increased toxicity to the cells [40]. After adding resazurin to the protoplast solution, incubation allows the cell to metabolize the resazurin. We found an incubation time of 3 h at 22 °C was optimal for our system. In contrast, there is a report that an incubation time of 1 h provided the best linear relationship between percentage reduction and cell density of tomato suspension cells, with reduction decreasing after 3–5 h [22]. Another study confirmed that the dynamic range of the resazurin assay (regarding the number of seeded cells, the initial concentration of resazurin, the type of readout, and the assay duration) needs to be optimized for each cell type, treatment type, and treatment duration [39]. Our study demonstrated that fluorescence provided higher sensitivity for detecting subtle differences in metabolic activity. Thus, determining and standardizing optimal conditions for each system is crucial for accurate measurements.

Testing nanoparticle cytotoxicity is crucial for safety assessments, but potential interference with fluorescent assays must be carefully managed. Nanoparticles can interact with assay reagents, potentially altering the chemical reactions and affecting the readout, leading to false results [15, 41]. To address these challenges, we included an interference control consisting of only assay buffers with nanoparticles and resazurin, but without protoplasts. No significant difference was observed compared to the blank control (assay buffers incubated with resazurin without protoplasts), indicating that the nanoparticles did not interfere with resazurin fluoresence.

A test compound is deemed cytotoxic if it causes at least a 20% reduction in signal, shows a concentration-dependent effect, and produces reproducible results across multiple experiments [27]. Our results indicate that Chol-but NE was cytotoxic regardless of concentration (Fig. 6a), likely due to cell membrane disruption caused by the hydrophobic interaction between membrane lipids and hydrophobic cholesterol butyrate. In contrast, LNPs were cytotoxic to canola protoplasts only at higher concentrations (Fig. 6b). The LNPs contain a cationic lipid, which may disrupt the cell membrane through electrostatic and hydrophobic interactions. The degree of disruption depends on the concentration of the LNPs. Similarly, standard and lab-synthesized SiO_2_ NS did not exhibit significant cytotoxicity at low concentrations (Fig. 8b and c). At higher concentrations, toxicity increased due to enhanced interactions between the nanoparticles and protoplast membranes, leading to greater internalization, potential membrane rupture, and cell death [42, 43]. In contrast, Ag NS with citrate significantly reduced protoplasts viability even at a low concentration of 5 ng/ml (Fig. 8a). Previous research has shown that Ag NSs significantly elevate ROS levels, leading to oxidative stress and subsequent cytotoxicity in plant cells [1, 2].

In our study, the lab-synthesized SiO_2_ NS (230 nm) with a high negative zeta potential (-56.9 mV) exhibited lower toxicity to plant protoplasts at high concentrations compared to smaller commercial SiO_2_ NS (12 nm) with a lower negative zeta potential (-48.1 mV) (Table 1; Fig. 8c). Despite both SiO_2_ NSs having zeta potentials with magnitudes greater than the threshold value of 20 or 30 mV [42], the observed toxicity is likely attributed to the increased surface area and higher penetration capability of the smaller nanoparticles. This supports previous findings that nanoparticle size and surface charge significantly affect toxicity and cellular uptake [43, 44]. For Ag NS, those with a higher zeta potential (-55.4 mV, 100 nm) exhibited higher toxicity compared to smaller nanospheres (-43 mV, 20 nm) at the same concentrations (Table 1; Fig. 8a). The larger Ag NSs contain higher amount of silver, which can lead to increased ROS levels, resulting in oxidative stress and cytotoxicity in plant cells [1, 2]. Conversely, the standard SiO_2_ NS with a medium size (50 nm) and a higher zeta potential (-45.8 mV) exhibited lower toxicity compared to both smaller and larger SiO_2_ NSs with lower zeta potentials (Table 1; Fig. 8b). The specific cause of the unexpected effects of size and zeta potential on cytotoxicity remains unclear. Similar findings in other studies have linked these variations to differences in fabrication methods [45–47]. These results indicate that nanoparticle size and zeta potential are crucial in determining toxicity, emphasizing the need to consider these factors in evaluating nanoparticle safety for plant cells.

## Conclusion

Despite recent advances in employing nanomaterials for gene delivery across biological systems, a method to screen nanomaterial cytotoxicity in protoplasts is still lacking. Given that metabolic activity indicates cytotoxicity, we developed an assay for precise measurement of protoplast metabolic activity. In this work, we evaluated a resazurin assay for the rapid quantification of canola protoplasts viability incubated with different nanoparticles. The resazurin assay offers a precise and efficient method for assessing canola protoplast viability, enabling a more accurate analysis of nanomaterial effects on protoplasts. The versatility and scalability of this approach make it a valuable tool for screening nanomaterial cytotoxicity before using nanomaterials for genetic engineering of protoplasts.

## Electronic supplementary material

Below is the link to the electronic supplementary material.

Supplementary Material 1: A glass microscope slide of a hemocytometer showing trypan blue aggregation formed in W5 and MMG Buffers. (**a**) Formation of trypan blue aggregation in W5 (upper grid area) and MMG buffer (lower grid area). (**b**) Chemical structure of trypan blue illustrating the interaction of calcium and magnesium ions with the sulfonate groups, leading to aggregate formation. 10 µl of each buffer was mixed with 10 µl of trypan blue and loaded on a hemocytometer

Supplementary Material 2: Zeta potential and particle size distribution of nanoparticles measured by dynamic light scattering (DLS)

## Data Availability

All data generated or analysed during this study are included in this published article [and its additional information files].

## Abbreviations

ROS: Reactive oxygen species
MES: 2-Morpholinoethanesulphonic acid
BSA: Bovine serum albumin
DMSO: Dimethyl sulfoxide
SiO_2_ NS: Silica nanosphere
Ag NS: Silver nanospheres
TEOS: Tetraethoxysilane
DODMA: 2-Dioleyloxy-N, N-Dimethyl-3-Aminopropane
LNP: Lipid nanoparticle
Chol-but NE: Cholesteryl butyrate nanoemulsion
DLS: Dynamic Light Scattering
SEM: Scanning Electron Microscopy

## References

[1] Nair PMG, Chung IM. Physiological and molecular level effects of silver nanoparticles exposure in rice (Oryza sativa L.) seedlings. Chemosphere. 2014;112:105–13. 25048895 10.1016/j.chemosphere.2014.03.056

[2] Geisler-Lee J, Wang Q, Yao Y, Zhang W, Geisler M, Li K, et al. Phytotoxicity, accumulation and transport of silver nanoparticles by Arabidopsis thaliana. Nanotoxicology. 2012;7(3):323–37. 22263604 10.3109/17435390.2012.658094

[3] Sembada AA, Lenggoro IW. Transport of nanoparticles into plants and their detection methods. Nanomaterials (Basel). 2024;14(2):131. 10.3390/nano14020131 PMC10819755 38251096

[4] Ali S, Mehmood A, Khan N. Uptake, translocation, and consequences of nanomaterials on plant growth and stress adaptation. J Nanomaterials. 2021:6677616.

[5] Giorgetti L, Spanò C, Muccifora S, Bottega S, Barbieri F, Bellani L, et al. Exploring the interaction between polystyrene nanoplastics and Allium cepa during germination: internalization in root cells, induction of toxicity and oxidative stress. Plant Physiol Biochem. 2020;149:170–7. 32070910 10.1016/j.plaphy.2020.02.014

[6] Lian J, Wu J, Xiong H, Zeb A, Yang T, Su X, et al. Impact of polystyrene nanoplastics (PSNPs) on seed germination and seedling growth of wheat (Triticum aestivum L). J Hazard Mater. 2020;385:121620. 10.1016/j.jhazmat.2019.121620 31744724

[7] Siddiqi KS, Husen A. Plant response to engineered metal oxide nanoparticles. Nanoscale Res Lett. 2017;12(1):92. 10.1186/s11671-017-1861-y PMC5293712 28168616

[8] Badaró Costa NL, Carvalho CR, Clarindo WR. Improved procedures to assess plant protoplast viability: evidencing cytological and genomic damage. Cytologia (Tokyo). 2018;83(4):397–405.

[9] Kuzminsky E, Meschini R, Terzoli S, Pavani L, Silvestri C, Choury Z, et al. Isolation of mesophyll protoplasts from mediterranean woody plants for the study of DNA integrity under abiotic stress. Front Plant Sci. 2016;7:1168. 10.3389/fpls.2016.01168 PMC4983556 27574524

[10] Aoyagi H. Application of plant protoplasts for the production of useful metabolites. Biochem Eng J. 2011;56:1–8.

[11] Quent VMC, Loessner D, Friis T, Reichert JC, Hutmacher DW. Discrepancies between metabolic activity and DNA content as tool to assess cell proliferation in cancer research. J Cell Mol Med. 2010;14(4):1003–13. 20082656 10.1111/j.1582-4934.2010.01013.x PMC3823131

[12] Konopka K, Pretzer E, Felgner PL, Düzgüneş N. Human immunodeficiency virus type-1 (HIV-1) infection increases the sensitivity of macrophages and THP-1 cells to cytotoxicity by cationic liposomes. Biochim Biophys Acta Mol Cell Res. 1996;1312(3):186–96. 10.1016/0167-4889(96)00033-x 8703987

[13] Baker CN, Banerjee SN, Tenover FC. Evaluation of alamar colorimetric MIC method for antimicrobial susceptibility testing of Gram-negative bacteria. J Clin Microbiol. 1994;32(5):1261–7. 10.1128/jcm.32.5.1261-1267.1994 PMC263664 8051254

[14] Chiaraviglio L, Kirby JE. Evaluation of impermeant, DNA-binding dye fluorescence as a real-time readout of eukaryotic cell toxicity in a high throughput screening format. Assay Drug Dev Technol. 2014;12(4):219–28. 24831788 10.1089/adt.2014.577 PMC4026211

[15] Rampersad SN. Multiple applications of alamar blue as an indicator of metabolic function and cellular health in cell viability bioassays. Sens (Switzerland). 2012;12(9):12347–60. 10.3390/s120912347 PMC3478843 23112716

[16] Zhang Y, Kao PL, Rampal A, Mafu S, Savinov S, Ma LJ. High-throughput screening assays to identify Plant natural products with antifungal properties against Fusarium oxysporum. In: Coleman J, editor. Methods in molecular biology. Humana Press Inc.; 2022. pp. 171–84. 10.1007/978-1-0716-1795-3_14 PMC9022449 34686985

[17] Uzarski JS, DiVito MD, Wertheim JA, Miller WM. Essential design considerations for the resazurin reduction assay to noninvasively quantify cell expansion within perfused extracellular matrix scaffolds. Biomaterials. 2017;129:163–75. 28343003 10.1016/j.biomaterials.2017.02.015 PMC5765551

[18] Ahmed SA, Gogal RM, Walsh JE. A new rapid and simple non-radioactive assay to monitor and determine the proliferation of lymphocytes: an alternative to [3H]thymidine incorporation assay. J Immunol Methods. 1994;170(2):211–24 10.1016/0022-1759(94)90396-4 8157999

[19] Gloeckner H, Jonuleit T, Lemke HD. Monitoring of cell viability and cell growth in a hollow-fiber bioreactor by use of the dye Alamar Bluee. J Immunol Methods. 2001;252(1-2):131–8. 10.1016/s0022-1759(01)00347-7 11334972

[20] Collins LA, Franzblau SG. Microplate Alamar Blue Assay versus BACTEC 460 system for high-throughput screening of compounds against Mycobacterium tuberculosis and Mycobacterium avium. Antimicrob Agents Chemother. 1997;41(5):1004–9. 10.1128/aac.41.5.1004 PMC163841 9145860

[21] Pfaller MA, Grant C, Morthland V, Rhine-Chalberg J. Comparative evaluation of alternative methods for broth dilution susceptibility testing of Fluconazole against Candida albicans. J Clin Microb. 1994;32(2):506–9. 10.1128/jcm.32.2.506-509.1994 PMC263062 8150963

[22] Byth HA, Mchunu BI, Dubery IA, Bornman L. Assessment of a simple, non-toxic Alamar Blue cell survival assay to monitor tomato cell viability. Phytochem Anal. 2001;12(5):340–6. 11705263 10.1002/pca.595

[23] De Fries R, Mitsuhashi M. Quantification of mitogen induced human lymphocyte proliferation: comparison of alamarBIueTM assay to 3H-thymidine incorporation assay. J Clin Lab Anal. 1995;9(2):89–95. 10.1002/jcla.1860090203 7714668

[24] Karuppusamy S, Rajasekaran KM. High Throughput Antibacterial screening of plant extracts by Resazurin Redox with Special Reference to Medicinal plants of western ghats. Global J Pharmacol. 2009;3(2):63–8.

[25] Barua P, You MP, Bayliss K, Lanoiselet V, Barbetti MJ. A rapid and miniaturized system using Alamar blue to assess fungal spore viability: implications for biosecurity. Eur J Plant Pathol. 2017;148(1):139–50.

[26] Birdi T, D’souza D, Tolani M, Daswani P, Nair V, Tetali P, et al. Assessment of the activity of selected Indian medicinal plants against Mycobacterium tuberculosis: a preliminary Screening using the Microplate Alamar Blue Assay. Eur J Med Plants. 2012;2(4):308–23.

[27] Longhin EM, El Yamani N, Rundén-Pran E, Dusinska M. The alamar blue assay in the context of safety testing of nanomaterials. Front Toxicol. 2022;4:981701. 10.3389/ftox.2022.981701 PMC9554156 36245792

[28] Li X, Sandgrind S, Moss O, Guan R, Ivarson E, Wang ES, et al. Efficient protoplast regeneration protocol and CRISPR/Cas9-mediated editing of glucosinolate transporter (GTR) genes in rapeseed (Brassica napus L). Front Plant Sci. 2021;12:680859. 10.3389/fpls.2021.680859 PMC8294089 34305978

[29] Silva AT, Nguyen A, Ye C, Verchot J, Ho Moon J. Conjugated polymer nanoparticles for effective siRNA delivery to tobacco BY-2 protoplasts. BMC Plant Biol. 2010;10(1):1–14. 10.1186/1471-2229-10-291 PMC3023792 21192827

[30] Yoo SD, Cho YH, Sheen J. Arabidopsis mesophyll protoplasts: a versatile cell system for transient gene expression analysis. Nat Protoc. 2007;2(7):1565–72. 17585298 10.1038/nprot.2007.199

[31] Stober W, Fink A, Ernst Bohn D. Controlled growth of Monodisperse silica spheres in the micron size range. J Colloid Interface Sci. 1968;26(1):62–9.

[32] Alam SB, Wang F, Qian H, Kulka M. Apolipoprotein C3 facilitates internalization of cationic lipid nanoparticles into bone marrow-derived mouse mast cells. Sci Rep. 2023;13(1):431. 10.1038/s41598-022-25737-7 PMC9828384 36624108

[33] Minelli R, Serpe L, Pettazzoni P, Minero V, Barrera G, Gigliotti CL, et al. Cholesteryl butyrate solid lipid nanoparticles inhibit the adhesion and migration of colon cancer cells. Br J Pharmacol. 2012;166(2):587–601. 22049973 10.1111/j.1476-5381.2011.01768.x PMC3417491

[34] Sheen J. Signal transduction in maize and Arabidopsis mesophyll protoplasts. Plant Physiology. Am Soc Plant Biol. 2001;127(4):1466–75. PMC1540179 11743090

[35] Torney F, Trewyn BG, Lin VSY, Wang K. Mesoporous silica nanoparticles deliver DNA and chemicals into plants. Nat Nanotechnol. 2007;2(5):295–300. 18654287 10.1038/nnano.2007.108

[36] Rouan D, Montan MH, Alibert G, Teissi J. Relationship between protoplast size and critical field strength in protoplast electropulsing and application to reliable DNA uptake in Brassica. Plant Cell Rep. 1991;10(3):139–43. 10.1007/BF00232045 24221493

[37] Marshall N, Goodwin CJ, Holt SJ. A critical assessment of the use of microculture tetrazolium assays to measure cell growth and function. Growth Regul. 1995;5(2):69–84. 7627094

[38] O’Brien J, Wilson I, Orton T, Pognan F. Investigation of the Alamar Blue (resazurin) fluorescent dye for the assessment of mammalian cell cytotoxicity. Eur J Biochem. 2000;267(17):5421–6. 10951200 10.1046/j.1432-1327.2000.01606.x

[39] Lavogina D, Lust H, Tahk MJ, Laasfeld T, Vellama H, Nasirova N, et al. Revisiting the resazurin-based sensing of cellular viability: widening the application Horizon. Biosens (Basel). 2022;12(4):196. 10.3390/bios12040196 PMC9032648 35448256

[40] Nociari MM, Shalev A, Benias P, Russo C. A novel one-step, highly sensitive fluorometric assay to evaluate cell-mediated cytotoxicity. J Immunol Methods. 1998;213(2):157–67. 10.1016/s0022-1759(98)00028-3 9692848

[41] Guadagnini R, Moreau K, Hussain S, Marano F, Boland S. Toxicity evaluation of engineered nanoparticles for medical applications using pulmonary epithelial cells. Nanotoxicology. 2015;9(S1):25–32. 24286383 10.3109/17435390.2013.855830

[42] Hu P, An J, Faulkner MM, Wu H, Li Z, Tian X, et al. Nanoparticle charge and Size Control Foliar Delivery Efficiency to Plant Cells and Organelles. ACS Nano. 2020;14(7):7970–86. 32628442 10.1021/acsnano.9b09178

[43] Zhi H, Zhou S, Pan W, Shang Y, Zeng Z, Zhang H. The promising nanovectors for gene delivery in plant genome engineering. Int J Mol Sci. 2022;23(15):8501. 10.3390/ijms23158501 PMC9368765 35955636

[44] Demirer GS, Zhang H, Matos JL, Goh NS, Cunningham FJ, Sung Y, et al. High aspect ratio nanomaterials enable delivery of functional genetic material without DNA integration in mature plants. Nat Nanotechnol. 2019;14(5):456–64. 30804481 10.1038/s41565-019-0382-5 PMC10461892

[45] Pierrat P, Wang R, Kereselidze D, Lux M, Didier P, Kichler A, et al. Efficient invitro and invivo pulmonary delivery of nucleic acid by carbon dot-based nanocarriers. Biomaterials. 2015;51:290–302. 25771019 10.1016/j.biomaterials.2015.02.017

[46] Liu M, Yang B, Deng F, Ji J, Yang Y, Huang Z, et al. Luminescence tunable fluorescent organic nanoparticles from polyethyleneimine and maltose: facile preparation and bioimaging applications. RSC Adv. 2014;4(43):22294–8.

[47] Sachdev A, Matai I, Gopinath P. Implications of surface passivation on physicochemical and bioimaging properties of carbon dots. RSC Adv. 2014;4(40):20915–21.

