# Supplementary figures and images for: Optimization of a rapid, sensitive, and high throughput molecular sensor to measure canola protoplast respiratory metabolism as a means of screening nanomaterial cytotoxicity
Source: Plant Methods. 2024 Oct 30;20:165. doi: 10.1186/s13007-024-01289-x (PMC11523603; doi:10.1186/s13007-024-01289-x)

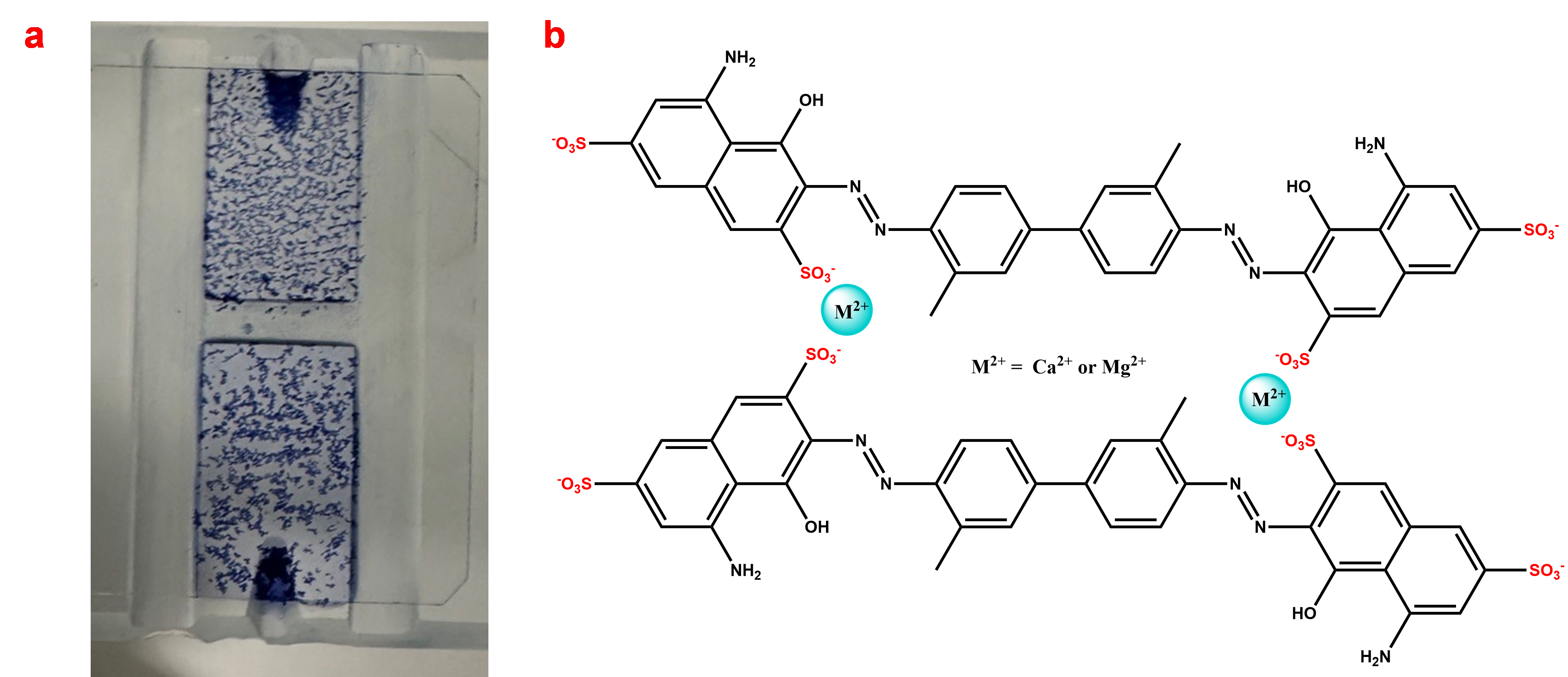

Supplement: Supplementary file 1 — Supplementary Material 1: A glass microscope slide of a hemocytometer showing trypan blue aggregation formed in W5 and MMG Buffers. (a) Formation of trypan blue aggregation in W5 (upper grid area) and MMG buffer (lower grid area). (b) Chemical structure of trypan blue illustrating the interaction of calcium and magnesium ions with the sulfonate groups, leading to aggregate formation. 10 µl of each buffer was mixed with 10 µl of trypan blue and loaded on a hemocytometer [file 13007_2024_1289_MOESM1_ESM.tif]
