# Supplementary material for: Optimization of a rapid, sensitive, and high throughput molecular sensor to measure canola protoplast respiratory metabolism as a means of screening nanomaterial cytotoxicity
Source: Plant Methods. 2024 Oct 30;20:165. doi: 10.1186/s13007-024-01289-x (PMC11523603; doi:10.1186/s13007-024-01289-x)
Supplement: Supplementary file 2 — Supplementary Material 2: Zeta potential and particle size distribution of nanoparticles measured by dynamic light scattering (DLS) [file 13007_2024_1289_MOESM2_ESM.docx]

| **Silica nanospheres; standard** | | |
| --- | --- | --- |
| **DLS** | 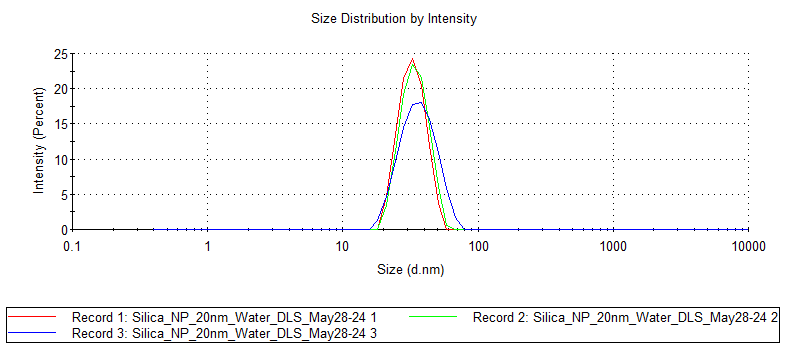 | 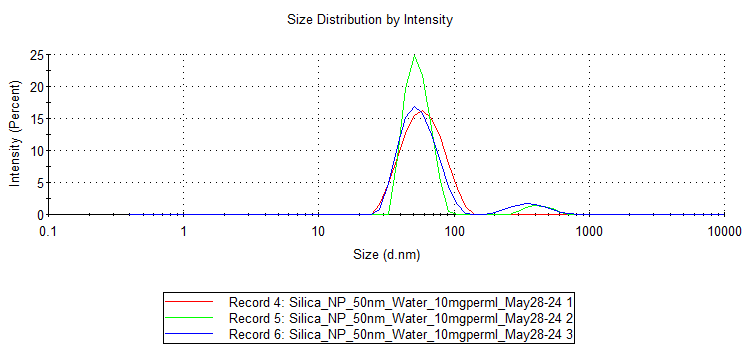 |
|  | **20 nm, 5mg/ml** | **50 nm, 10mg/ml** |
|  | 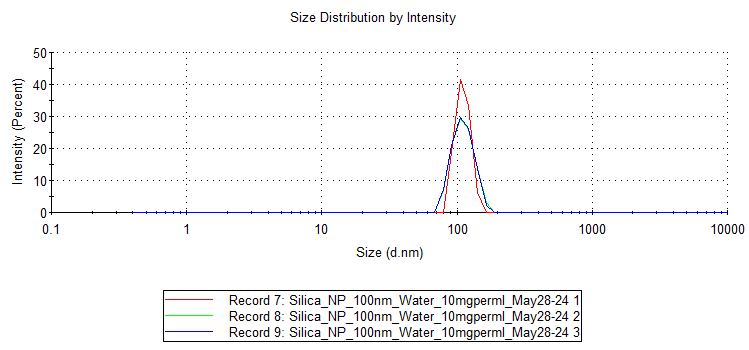 |  |
|  | **100 nm, 10 mg/ml** |  |
| **Zeta**  **Potential** | 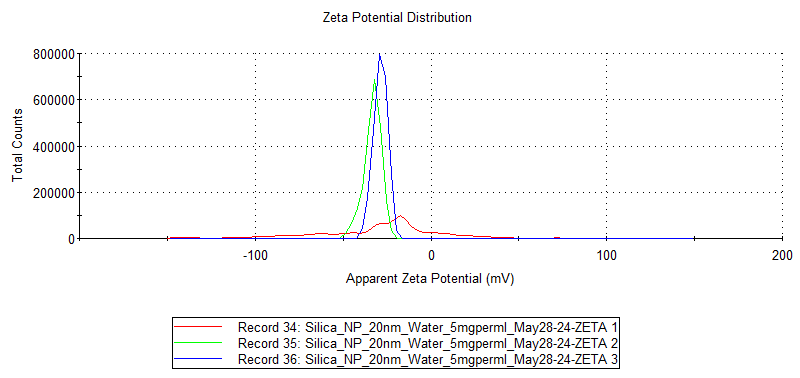 | 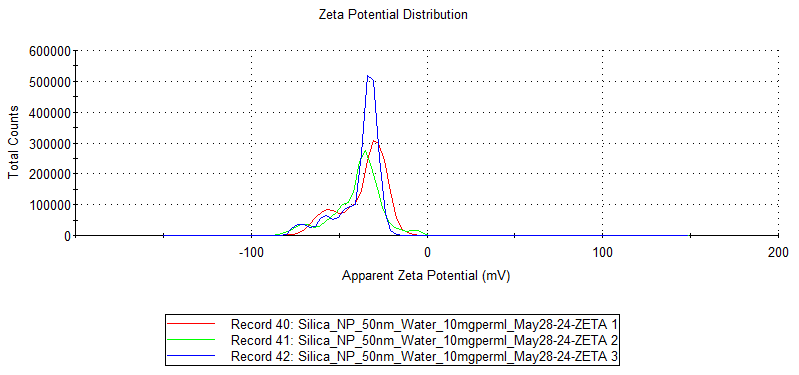 |
|  | **20 nm, 5mg/ml** | **50 nm, 10mg/ml** |
|  | 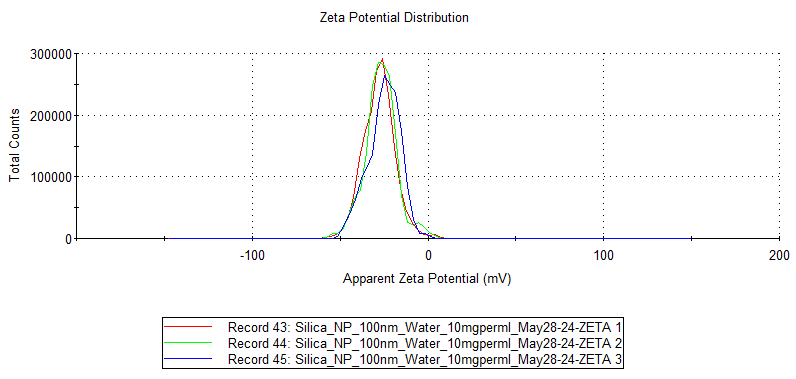 |  |
|  | **100 nm, 10 mg/ml** |  |
| **Silver nanospheres; citrate** | | |
| **DLS** | 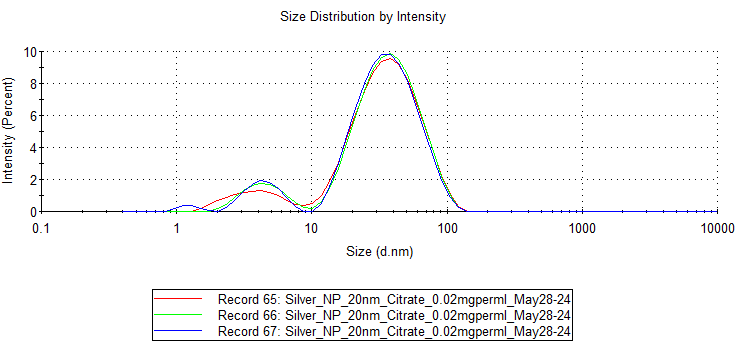 | 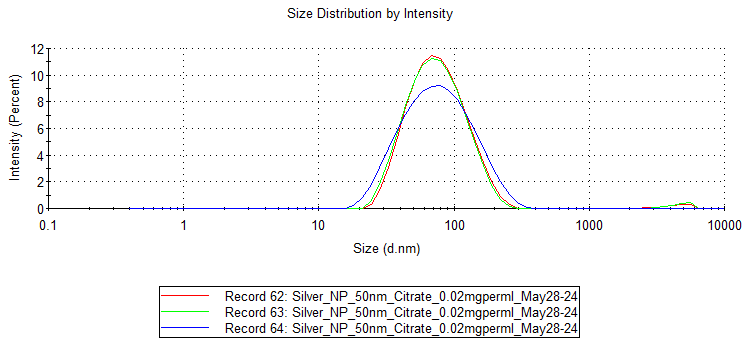 |
|  | **20 nm, 0.02mg/ml** | **50 nm, 0.02mg/ml** |
|  | 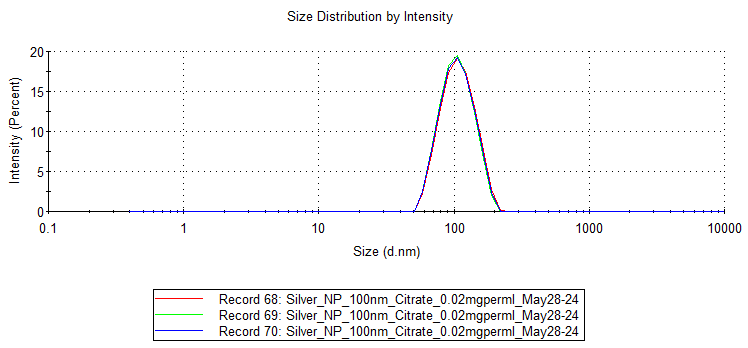 |  |
|  | **100 nm, 0.02mg/ml** |  |
| **Zeta Potential** | 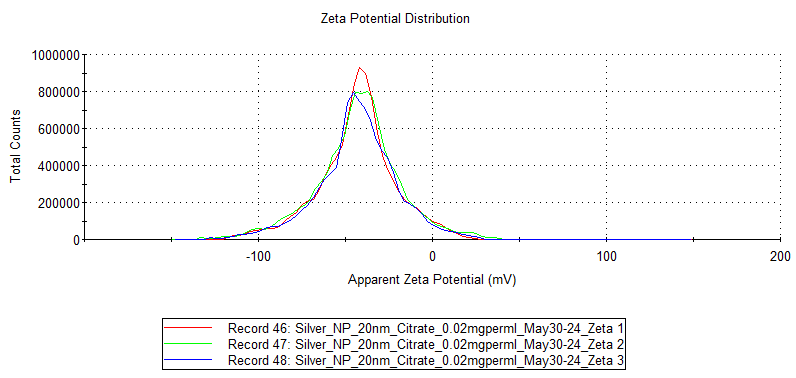 | 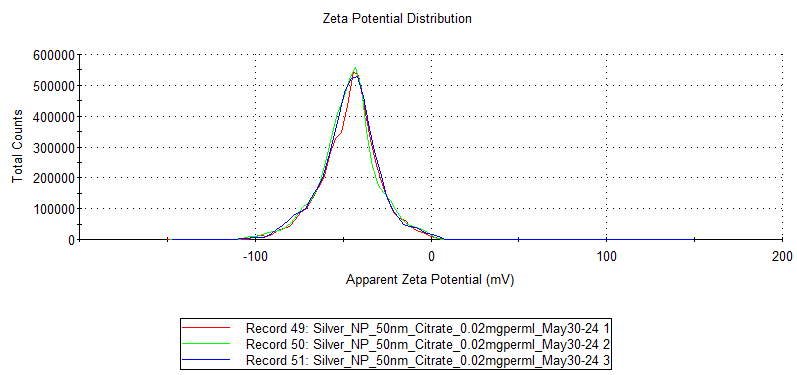 |
|  | **20 nm, 0.02mg/ml** | **50 nm, 0.02mg/ml** |
|  | 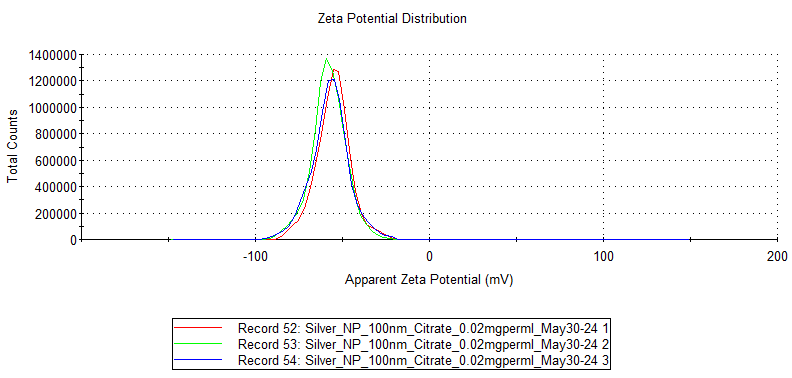 |  |
|  | **100 nm, 0.02mg/ml** |  |
| **Zeta Potential** | **Silver nanospheres (SiO_2_)** | |
|  |  |  |
|  | **12 nm, 1 mg/ml** | **40 nm, 1 mg/ml** |
|  |  |  |
|  | **230 nm, 1 mg/ml** |  |
